# Sphingosine-1-phosphate promotes erythrocyte glycolysis and oxygen release for adaptation to high-altitude hypoxia

**DOI:** 10.1038/ncomms12086

**Published:** 2016-07-15

**Authors:** Kaiqi Sun, Yujin Zhang, Angelo D'Alessandro, Travis Nemkov, Anren Song, Hongyu Wu, Hong Liu, Morayo Adebiyi, Aji Huang, Yuan E. Wen, Mikhail V. Bogdanov, Alejandro Vila, John O'Brien, Rodney E. Kellems, William Dowhan, Andrew W. Subudhi, Sonja Jameson-Van Houten, Colleen G. Julian, Andrew T. Lovering, Martin Safo, Kirk C. Hansen, Robert C. Roach, Yang Xia

**Affiliations:** 1 Department of Biochemistry and Molecular Biology, The University of Texas Health Science Center at Houston, Houston, Texas 77030, USA; 2 Graduate School of Biomedical Sciences, Biochemistry and Molecular Biology Program, The University of Texas Health Science Center at Houston, Houston, Texas 77030, USA; 3 Department of Biochemistry and Molecular Genetics, University of Colorado School of Medicine, Aurora, Colorado 80045, USA; 4 Department of Ophthalmology and Visual Science, The University of Texas Health Science Center at Houston, Houston, Texas 77030, USA; 5 Altitude Research Center, Department of Emergency Medicine, University of Colorado School of Medicine, Aurora, Colorado 80045, USA; 6 Department of Human Physiology, University of Oregon, Eugene, Oregon 97403, USA; 7 Department of Medicinal Chemistry, Virginia Commonwealth University, Richmond, Virginia 23298, USA; 8 Department of Nephrology, Xiangya Hospital, Central South University, Changsha, Hunan 410008, China

## Abstract

Sphingosine-1-phosphate (S1P) is a bioactive signalling lipid highly enriched in mature erythrocytes, with unknown functions pertaining to erythrocyte physiology. Here by employing nonbiased high-throughput metabolomic profiling, we show that erythrocyte S1P levels rapidly increase in 21 healthy lowland volunteers at 5,260 m altitude on day 1 and continue increasing to 16 days with concurrently elevated erythrocyte sphingonisne kinase 1 (Sphk1) activity and haemoglobin (Hb) oxygen (O_2_) release capacity. Mouse genetic studies show that elevated erythrocyte Sphk1-induced S1P protects against tissue hypoxia by inducing O_2_ release. Mechanistically, we show that intracellular S1P promotes deoxygenated Hb anchoring to the membrane, enhances the release of membrane-bound glycolytic enzymes to the cytosol, induces glycolysis and thus the production of 2,3-bisphosphoglycerate (2,3-BPG), an erythrocyte-specific glycolytic intermediate, which facilitates O_2_ release. Altogether, we reveal S1P as an intracellular hypoxia-responsive biolipid promoting erythrocyte glycolysis, O_2_ delivery and thus new therapeutic opportunities to counteract tissue hypoxia.

## Abstract (web summary)

The presence of the signalling lipid Sphingosine 1-phosphate (S1P) in erythrocytes has unclear physiological implications. Here the authors show that the S1P-generating enzyme Sphingosine kinase type 1 and its product S1P play an important role in the red blood cell adaptation to hypoxic environments in mice and humans.

## Graphical abstract

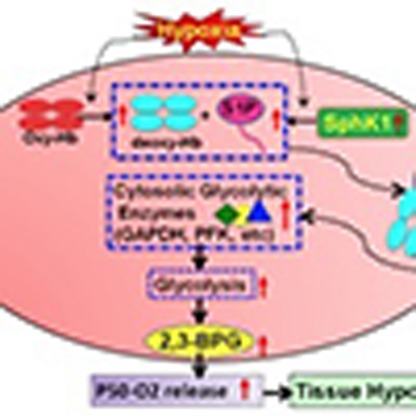

Spingosine 1-phosphate (S1P), a bioactive signalling lipid^1^, is generated by two enzymes, sphingosine kinase 1 (Sphk1), localized in the cytosol^2^, and sphingosine kinase 2 (SphK2), mainly residing in the nucleus^3^. S1P is quickly degraded by specific phosphatases or lyases localized in the endoplasmic reticulum (ER)^4^,^5^,^6^. Mature red blood cells (RBCs) contain Sphk1 but no S1P degrading enzymes, perhaps due to the lack of nuclei and ER^7^,^8^. Because of such unique features, erythrocytes produce and store large amounts of S1P^9^, which accounts for nearly all embryonic S1P and ∼75% of adult plasma S1P in mice^10^,^11^. Substantial studies demonstrated that extracellular S1P is involved in many physiological activities including immune cell trafficking^12^, hematopoietic stem cell trafficking^13^, vascular integrity^14^ and cell proliferation^15^ by activating its five G-protein-coupled receptors. In addition, intracellular S1P also regulates histone deacetylation^16^ and ubiquitin functions^17^. However, since the initial discovery of the enrichment of S1P in erythrocytes, it is still unknown whether erythrocytes merely supply the plasma with S1P or utilize S1P for their own purpose.

As the only cell type responsible for delivering oxygen (O_2_), RBCs quickly respond to hypoxia, defined as inadequate O_2_ supply to the whole body or a region of the body, by increasing their O_2_ delivery ability. Hypoxia frequently occurs in healthy individuals exposed to a low-O_2_-content environment, such as places at high altitude^18^,^19^. It is widely noticed that people differ in the ability to adapt to high-altitude hypoxia^19^,^20^,^21^,^22^. Inability to quickly adapt to high-altitude hypoxia can result in pulmonary oedema, stroke, cardiovascular dysfunction and even death^18^,^23^,^24^. Hypoxia is also commonly seen in patients with cardiovascular^23^,^25^,^26^, respiratory^27^,^28^ and haemolytic diseases, which frequently promote multiple end-organ damage and failure. Thus, hypoxia is a dangerous condition for both normal individuals and patients with cardiovascular, respiratory and haemolytic diseases. However, there is an enormous gap in our understanding of the specific factors and signalling pathways involved in hypoxia adaptation and an even larger one in identifying strategies to reduce hypoxia-induced tissue damage.

To this end, we performed metabolomics profiling and functional analyses of erythrocytes of blood samples collected from 21 young and healthy lowland individuals at sea level and at 5,260 m, for up to 16 days (illustration in Fig. 1a). We showed that the S1P levels of RBCs were elevated in all lowland volunteers when brought to 5,260 m. Translating results from the human high-altitude study to a mouse model of hypoxia, we demonstrate that elevated RBCs' Sphk1 activity underlies increased S1P production within RBCs in hypoxia conditions and that elevated RBCs' S1P is beneficial to counteract tissue hypoxia independent of S1P receptors. Mechanistically, we further revealed that erythrocyte S1P is an important hypoxia-responsive biolipid functioning intracellularly to promote erythrocyte glycolysis and trigger O_2_ delivery. Thus, our findings revealed a previously unrecognized beneficial role and the underlying mechanisms of elevated erythrocyte S1P in hypoxia adaptation. These results may provide the basis for novel therapeutic possibilities to counteract hypoxic conditions.

## Results

### Altitude induces S1P levels and Sphk1 activity in human RBCs

**Figure 1:**
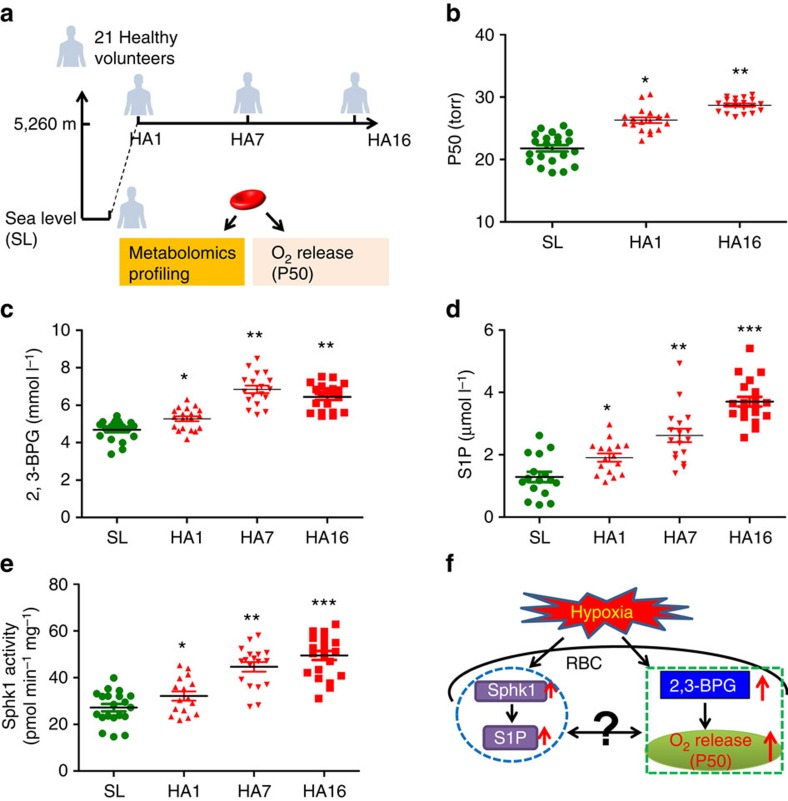
Concurrent increase of erythrocyte Sphk1 activity, S1P production and O_2_ delivery ability in human high-altitude study. (**a**) Schematic representation of human high-altitude study: nonbiased metabolomic profiling coupled with erythrocyte function analysis was performed on the erythrocytes isolated from 21 human volunteers at sea level (SL), and after 12 h (HA1), 7 days (HA7) and 16 days (HA16) at 5,260 m altitude. (**b**) Erythrocyte O_2_ release capacity was measured as P50. 2,3-BPG level (**c**) and S1P level (**d**) were quantified in human high-altitude samples. (**e**) Erythrocyte Sphk1 activity in human high-altitude samples. (**f**) Schematic representation showing concurrent increase of erythrocyte S1P metabolism with O_2_ delivery identified in human high-altitude study. Mean±s.e.m; *n*=16–21 per group; *P<0.05 versus SL, **P<0.01 versus HA1, ***P<0.01 versus HA7, two-way ANOVA.

In an effort to determine human erythrocyte response to hypoxia, we recruited 21 young and healthy lowland individuals and brought them to high altitude at 5,260 m for a total of 16 days. Nonbiased metabolomic profiling was performed on the erythrocytes isolated from those 21 human volunteers at sea level (SL), and after 12 h (HA1), 7 days (HA7) and 16 days (HA16) at 5,260 m altitude (for details, see Supplementary Data 1). In addition, we monitored erythrocyte O_2_ releasing capacity by measuring P50, the partial O_2_ pressure required to reach 50% Hb-O_2_ saturation, from human subjects at SL and at high altitude (illustration in Fig. 1a).

Consistent with the previous high-altitude studies^29^, we found that erythrocyte O_2_ releasing capacity measured by P50 was significantly increased by ∼20% as rapidly as 12 h at high altitude, and continued increasing to day 16 in human volunteers (Fig. 1b). Moreover, metabolomic profiling identified 233 metabolites in the erythrocytes (Supplementary Data 1), and showed that the levels of erythrocyte 2,3-bisphosphoglycerate (2,3-BPG), a specific allosteric modulator promoting O_2_ release from haemoglobin, increased in response to high-altitude hypoxia on day 1 and was maintained at high level until day 16, as quantified by spectrophotometric assays (Fig. 1c). Notably, our metabolomics profiling coupled with S1P level quantification revealed that erythrocyte S1P levels rapidly increased within 12 h at high altitude and further increased to approximately twofold on day 7 and threefold on day 16, consistent with the trends observed for 2,3-BPG and P50 (Fig. 1d). Sphk1 is the major enzyme responsible for the production of S1P in erythrocytes^11^. Reassuringly, we found that erythrocyte Sphk1 activity was significantly induced at high altitude like S1P (Fig. 1e). Consistent to the notion that the erythrocyte is the major cell source of circulating S1P (refs 10, 11), plasma S1P levels were also increased in humans after a 16-day stay at high altitude (Supplementary Fig. 1a). Thus, we demonstrated that Sphk1 activity and S1P levels are induced in mature human erythrocytes by high altitude.

### Sphk1 promotes O_2_ release from mouse RBCs to offset hypoxia

**Figure 2:**
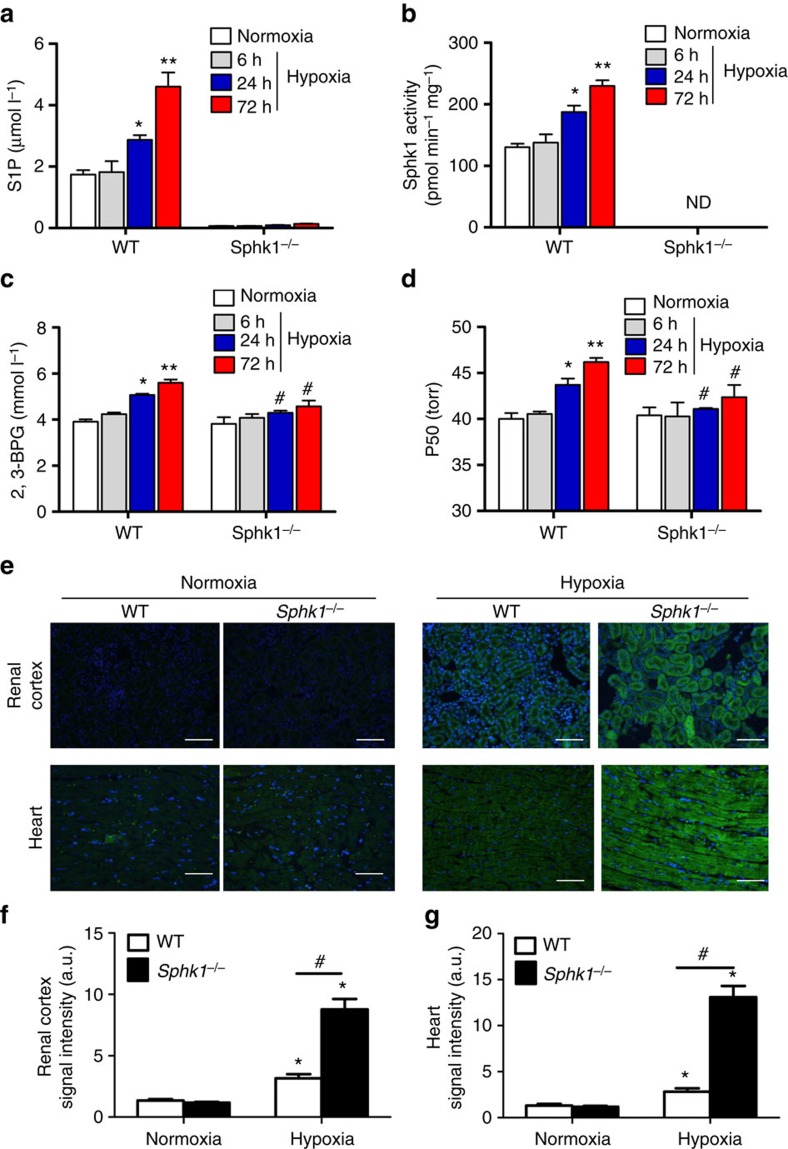
Hypoxia-induced Sphk1 and S1P increase regulate 2,3-BPG level, O_2_ delivery ability and tissue hypoxia in mice. Erythrocyte Sphk1 activity (**a**), S1P level (**b**), 2,3-BPG level (**c**) and P50 (**d**) in WT and *Sphk1*^*−/−*^ mice in normoxia and hypoxia for different treatment time. (**e**) Tissue hypoxia signals measured by Hypoxyprobe in renal cortex and heart in WT and *Sphk1*^*−/−*^ mice in normoxia and hypoxia for 72 h. Quantification of hypoxia signals in renal cortex (**f**) and heart (**g**). Mean±s.e.m; *n*=6–8 per treatment time point; *P<0.05 versus 6 h or normoxia, **P<0.05 versus 24 h, ^#^*P*<0.05 versus WT, Student's *t*-test and one-way ANOVA. Scale bar, 50 μM.

Since elevated 2,3-BPG levels are known to increase erythrocyte O_2_ release under hypoxia^29^, our findings raise an intriguing possibility that elevated erythrocyte Sphk1-mediated increase in S1P production could contribute to the induction of erythrocyte 2,3-BPG levels and increased O_2_ releasing capacity to adapt to high-altitude hypoxia (Fig. 1f). As it is difficult to determine in humans if elevated erythrocyte Sphk1-mediated S1P production under hypoxia plays a role to induce erythrocyte 2,3-BPG levels and increase O_2_ release to peripheral tissues, we, focused our further experimental efforts on mice. Briefly, we exposed wild-type (WT) and Sphk1-deficient (*Sphk1*^*−/−*^)^30^ mice to hypoxia (10% oxygen, close to oxygen level at 5,260 m altitude) for up to 72 h. Similar to the human high-altitude studies, we found that erythrocyte SphK1 activity and S1P levels increased in a time-dependent manner (Fig. 2a,b). Moreover, erythrocyte 2,3-BPG levels and P50 were significantly elevated in WT mice similar to our human studies (Fig. 2c,d). In contrast, Sphk1 activity is undetectable and erythrocyte S1P levels are only ∼1/50 that of WT mice in *Sphk1*^*−/−*^ mice. Moreover, hypoxia-mediated increase of erythrocyte S1P, 2,3-BPG and P50 were significantly impaired in *Sphk1*^*−/−*^ mice (Fig. 2b–e). Similar to our findings in humans, plasma S1P levels were induced by hypoxia in WT mice in a time-dependent manner but blunted in *Sphk1*^*−/−*^ mice (Supplementary Fig. 1b). These results indicate that elevated Sphk1-mediated S1P production is required for hypoxia-induced elevation of mouse erythrocyte 2,3-BPG levels and subsequent O_2_ releasing capacity.

Next, we assessed whether elevated Sphk1-mediated increase in S1P production and subsequent elevation of erythrocyte 2,3-BPG and P50 are beneficial to protect against tissue hypoxia using Hypoxyprobe. The kidneys are susceptible to hypoxia due to the parallel arrangement of arterial and venous preglomerular and postglomerular vessels, which allows oxygen to pass from arterioles into the postcapillary venous system via shunt diffusion^31^. The heart is also susceptible to hypoxia due to fast consumption of O_2_ for cardiac functions^26^. No Hypoxyprobe signals were detected in tissue sections of WT or *Sphk1*^*−/−*^ mice in normoxia (Fig. 2e). However, in hypoxia, immune-fluorescence (IF) analysis of the Hypoxyprobe signals showed slightly elevated staining in kidneys and hearts after 72 h in hypoxia compared with normoxia in WT mice (Fig. 2f). In contrast, severe hypoxia in kidney and heart was observed in *Sphk1*^*−/−*^ mice after 72 h in hypoxia (Fig. 2f). Image quantification analysis demonstrated that the intensity of Hypoxyprobe signals in the kidney and heart of *Sphk1*^*−/−*^ mice was more than twofold of that of WT mice (Fig. 2g,h). Since differences in pulmonary function in hypoxia could also affect O_2_ availability and thereby tissue hypoxia, we measured arterial Hb-O_2_ saturation (SaO_2_) to assess lung function in WT and *Sphk1*^*−/−*^ mice in normoxia and hypoxia. Although we observed a significant decrease of SaO_2_ in both WT and *Sphk1*^*−/−*^ mice in hypoxia, there were no significant differences between WT and *Sphk1*^*−/−*^ mice under either normoxia or hypoxia for up to 72 h (Supplementary Fig. 2), indicating that the increased Hypoxyprobe signals in the kidney and heart of *Sphk1*^*−/−*^ mice were not due to decreased lung uptake of O_2_ under hypoxia. Instead, our findings suggest that decreased erythrocyte S1P production in *Sphk1*^*−/−*^ mice led to reduced 2,3-BPG production and O_2_ releasing capacity and thus, to more kidney and heart hypoxia.

**Figure 3:**
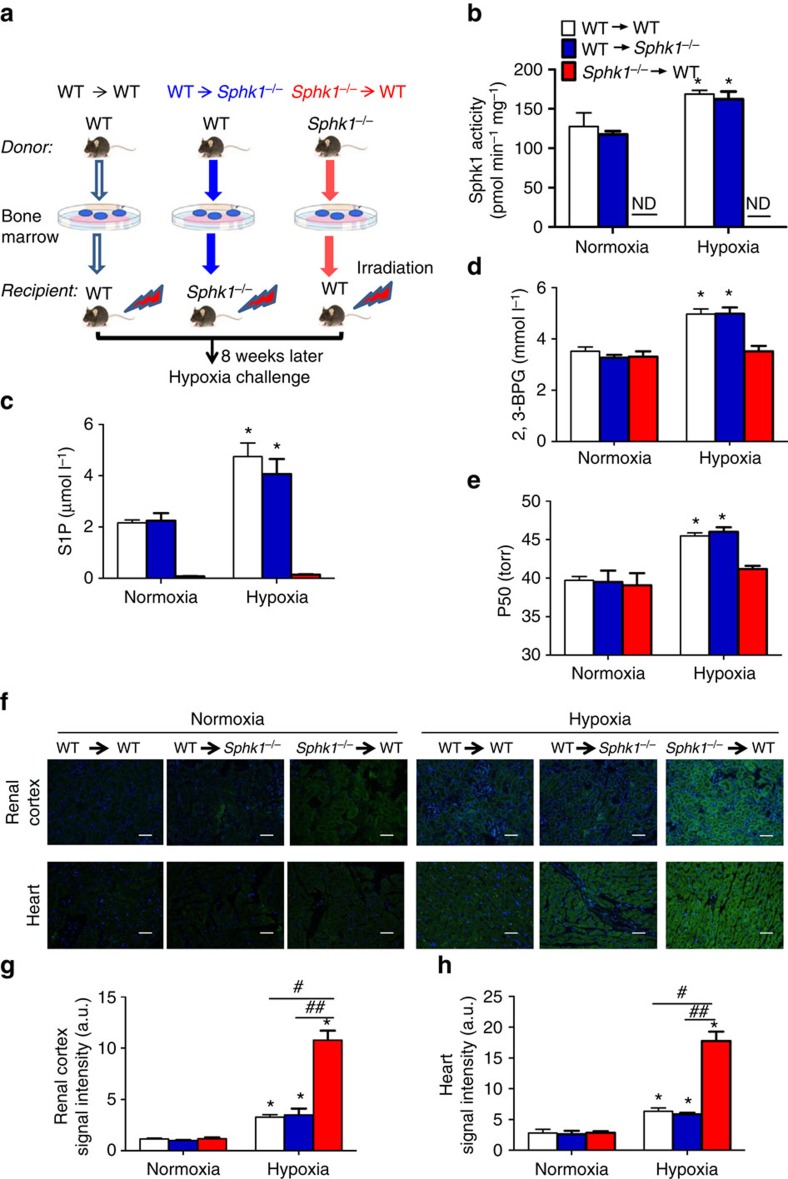
Bone marrow-derived Sphk1 and S1P are responsible for protecting tissue hypoxia by inducing erythrocyte 2,3-BPG levels and O_2_ release capacity. (**a**) Schematic illustration of reciprocal bone marrow transplantation (BMT) between WT and *Sphk1*^*−/−*^ mice. Erythrocyte Sphk1 activity (**b**), S1P (**c**), 2,3-BPG level (**d**) and P50 (**e**) in each group of mice in normoxia and hypoxia for different treatment times. (**f**) Tissue hypoxia signals measured by Hypoxyprobe in renal cortex and heart in each group of mice in normoxia and hypoxia for 72 h. Quantification of hypoxia signals in renal cortex (**g**) and heart (**h**). Mean±s.e.m; *n*=8 per group of mice; *P<0.05 versus normoxia, ^#^*P*<0.05 versus WT to WT, ^##^*P*<0.05 versus WT to *Sphk1*^*−/−*^, Student's *t*-test and one-way ANOVA. Scale bar, 50 μM.

Erythrocytes are derived from hematopoietic stem cells in the bone marrow and the vast majority of BM-derived cells in the circulation are erythrocytes. Thus, to determine if elevated erythrocyte Sphk1 activity-mediated increase in S1P is responsible for hypoxia adaptation to protect against tissue hypoxia, we conducted reciprocal bone marrow transplantation (BMT) between WT and *Sphk1*^*−/−*^ mice. Specifically, three groups of mice were generated by BMT including: (1) ‘*WT-to-Sphk1*^*−/−*^' *group* was designed to critically determine if Sphk1 expressed only in hematopoietic-derived cells could rescue severe tissue hypoxia in *Sphk1*^*−/−*^ mice transplanted with WT mouse BM; (2) ‘*Sphk1*^*−/−*^-to-*WT*' group was generated by transplanting BM of *Sphk1*^*−/−*^ mice to WT mice to examine if Sphk1 deficiency only in BM-derived cells is sufficient to cause severe tissue hypoxia; (3) ‘*WT*-to-*WT*' group is WT mouse BM transplanted to WT mice. Eight weeks after BMT, Sphk1 activity in mature erythrocytes was detected as an indicator of chimerism (Supplementary Fig. 3). Three groups of mice with more than 95% chimerism were subjected to hypoxia challenge for 72 h, respectively (illustration in Fig. 3a). As expected, the basal levels of erythrocyte Sphk1 activity in the ‘*WT*-to-*Sphk1*^*−/−*^' group were similar to the ‘*WT-to-WT*' mice, while it was undetectable in ‘*Sphk1*^*−/−*^-to-*WT*' mice as in the global *Sphk1*^*−/−*^ mice under normoxia condition, indicating successful BMT (Fig. 3b). Similarly, erythrocyte S1P levels in the ‘*WT*-to-*Sphk1*^*−/−*^' group were no different compared with the ‘*WT*-to-*WT*' mice, while it was 20-fold higher than that in ‘*Sphk1*^*−/−*^-to-*WT*' mice (Fig. 3c). Consistent to global knockouts, no obvious difference in erythrocyte 2,3-BPG and P50 was observed under normoxia condition (Fig. 3d,e). However, after 72- h hypoxia exposure, ‘*WT-to-Sphk1*^*−/−*^' group showed a 30% increase in Sphk1 activity, one-fold induction of S1P levels, 50% increase in 2,3-BPG levels and 6 torr elevation in P50 in the erythrocytes as with the *WT* to *WT* group (Fig. 3b–e). In contrast, Sphk1 activity was undetectable and 2,3-BPG and P50 were not induced by 72 h hypoxia in WT mice transplanted with *Sphk1*^*−/−*^ mouse BM (Fig. 3b–e). No obvious Hypoxyprobe signals were detected in those three groups of mice under normoxia (Fig. 3f). However, after 72-h hypoxia exposure, we observed severe hypoxia in the kidneys and hearts from ‘*Sphk1*^*−/−*^-to-*WT*' mice similar to global *Sphk1*^*−/−*^ mice, indicating that deficiency of Sphk1 in BM-derived cells is sufficient to mimic the severe tissue hypoxia as seen in global *Sphk1*^*−/−*^ mice. In contrast, the ‘*WT-to-Sphk1*^*−/−*^' group mice showed significantly less Hypoxyprobe signals in the kidneys and hearts compared with that of ‘*Sphk1*^*−/−*^-to-*WT*' mice after 72-h exposure to hypoxia (Fig. 3f). Further image quantification analysis demonstrated that the intensity of Hypoxyprobe signals in the kidneys and hearts was significantly reduced in the ‘*WT*-to-*Sphk1*^*−/−*^' group but increased in ‘*Sphk1*^*−/−*^-to-*WT*' mice (Fig. 3g,h). Thus, reciprocal BMT studies led us to conclude that Sphk1 in BM-derived cells but not in peripheral tissue is responsible for adaptation to hypoxia by inducing erythrocyte S1P production, 2,3-BPG levels and O_2_ release.

### Intracellular S1P underlies increased 2,3-BPG production

Next, we performed *in vitro* studies involving cultured erythrocytes to determine if elevated erythrocyte Sphk1 directly contributed to hypoxia-induced 2,3-BPG in erythrocytes. Consistent with human and mouse findings, hypoxia induced Sphk1 activity in the cultured erythrocytes from WT mice, but no detectable activity in cultured erythrocytes isolated from *Sphk1*^*−/−*^ mice under both normoxia and hypoxia condition (Supplementary Fig. 4a). Moreover, we found that hypoxia directly induced 2,3-BPG levels in cultured erythrocytes from WT mice but not *Sphk1*^*−/−*^ mice (Supplementary Fig. 4b,c), indicating that hypoxia-induced erythrocyte Sphk1 activity is required for 2,3-BPG production in mouse erythrocytes.

Next, to determine if extracellular S1P signalling via its surface receptors directly induces 2,3-BPG production in erythrocytes, we isolated erythrocytes from both WT and *Sphk1*^*−/−*^ mice and pretreated them with exogenous S1P up to 250 nmol l^−1^, which is high enough to activate all of five S1PRs^32^ but insufficient to increase intracellular S1P, under normoxia and hypoxia conditions. However, S1P pretreatment with up to 250 nmol l^−1^ failed to further increase 2,3-BPG levels in cultured erythrocytes from WT mice under either noxmoxia or hypoxia conditions (Supplementary Fig. 4b). Moreover, S1P pretreatment could not rescue the lack of 2,3-BPG induction under both normoxia and hypoxia condition in cultured erythrocytes isolated from *Sphk1*^*−/−*^ mice (Supplementary Fig. 4c). Thus, these studies provided direct evidence that hypoxia-induced Sphk1 activity-mediated elevation of S1P functions independently of S1P receptors to induce 2,3-BPG levels in the erythrocytes.

### Glycolysis is induced by high altitude in human RBCs

**Figure 4:**
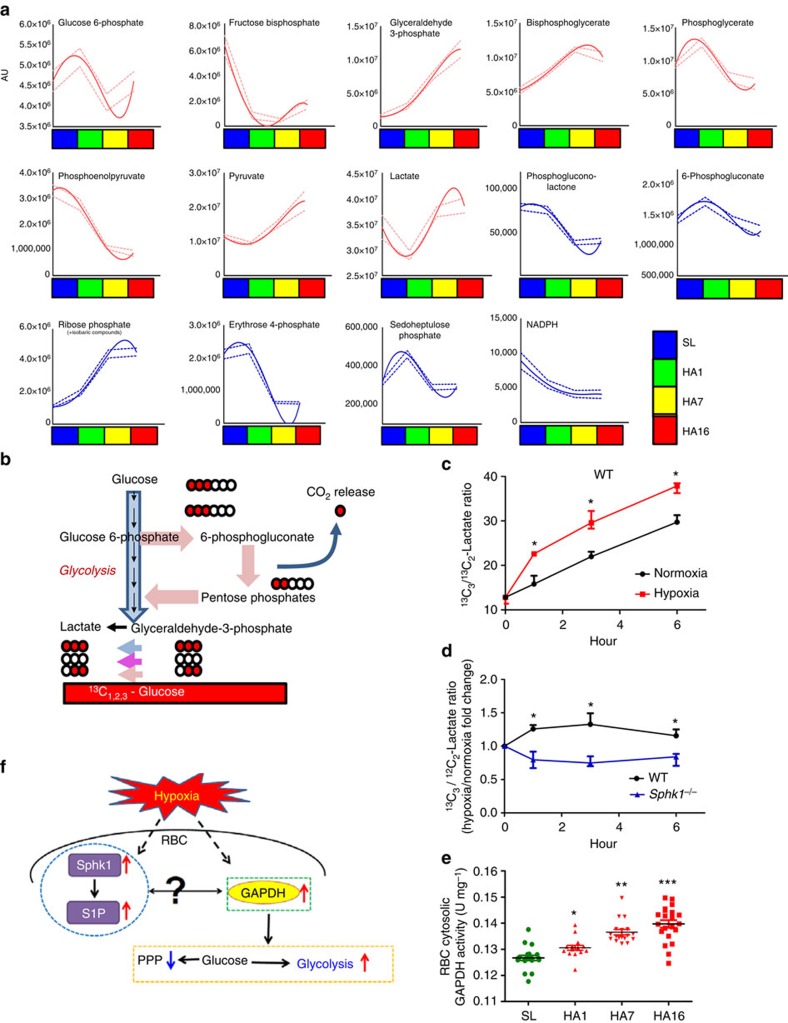
Alteration of erythrocyte glucose metabolism favouring glycolysis in hypoxia. (**a**) Metabolomic screening revealed time-dependent increase of glycolysis and decrease of PPP in erythrocytes from humans exposed to high-altitude hypoxia. (**b**) Schematic illustration of glucose metabolism flux detection using ^13^C_1,2,3_-glucose. (**c**) ^13^C_3_/^13^C_2_-lactate ratio determined in WT erythrocytes in normoxia and hypoxia. Fold change of ^13^C_3_/^13^C_2_-lactate ratio (**d**) in WT and *Sphk1*^*−/−*^ erythrocytes in hypoxia to normoxia. (**e**) Erythrocyte cytosolic GAPDH activity in erythrocyte from humans exposed to high-altitude hypoxia. (**f**) Schematic representation showing concurrent change of erythrocyte S1P metabolism with shift of glucose metabolism towards glycolysis in human high-altitude study. Mean±s.e.m. For human studies, *n*=16–21 per group; for mouse studies *n*=4 per group; *P<0.01 versus SL, normoxia or *Sphk1*^*−/−*^, **P<0.01 versus HA1, ***P<0.05 versus HA7, Student's *t*-test, two-way ANOVA and one-way ANOVA.

In an effort to determine the molecular basis underlying elevated S1P-mediated 2,3-BPG induction and O_2_ releasing capacity inside erythrocytes under hypoxia, we revisited our metabolomic profiling data. Intriguingly, our high throughput nonbiased metabolomics screening not only confirmed previous studies showing that 2,3-BPG levels were increased by high-altitude hypoxia (Fig. 1c), but also revealed that levels of representative glycolytic metabolite glyceraldehyde-3-phosphate (G3P) and upstream intermediate of 2,3-BPG, were significantly elevated in hypoxia after 12 h and continued to increase to day 16 (Fig. 4a). In contrast, all of the upstream intermediates of G3P, including glucose-6-phosphate and fructose 1,6-bisphosphate and the two most immediate intermediates downstream of 2,3-BPG including 2/3-phosphoglycerate and phosphoenolpyruvate, were significantly reduced in response to high-altitude hypoxia in a time-dependent manner. These findings suggest that the glycolytic pathway prior to shunting to the erythrocyte-specific Rapoport-Luebering Shunt, which is a diversion of main glycolytic pathway for the production of 2,3-BPG, is significantly induced (Fig. 4a).

There are two major glucose metabolism pathways in erythrocytes: the first one is the Embden–Meyerhof glycolytic pathway, which generates energy and glycolytic intermediates such as 2,3-BPG to promote O_2_ release; the second one is the pentose phosphate pathway (PPP), which produces reducing equivalents to regenerate nicotinamide adenine dinucleotide phosphate-dependent antioxidant glutathione and enzymes to protect against oxidative stress^33^. In support of our observation that induction of glycolytic pathways favours 2,3-BPG induction under hypoxia, we further showed that steady-state levels of PPP intermediates, such as phosphogluconolactone, 6-phosphogluconate, erythrose 4-phosphate and sedoheptulose phosphate (Fig. 4a), were significantly decreased in hypoxia compared with sea level. As such, we observed that reduced nicotinamide adenine dinucleotide phosphate (NADPH), the PPP derived reducing equivalent, was significantly decreased in high-altitude hypoxia in a time-dependent manner (Fig. 4a). Thus, our non-biased high throughput metabolomic profiling implicates that erythrocytes adapt to high-altitude hypoxia by enhancing glucose flux through glycolytic pathway and decreasing its flux through the PPP and thus facilitating 2,3-BPG production.

### RBC Sphk1 promotes glucose fluxes to glycolysis in hypoxia

We found that erythrocyte Sphk1 activity and 2,3-BPG levels were induced by hypoxia in healthy human subjects and WT mice, but not in *Sphk1*^*−/−*^ mice. Thus, it is possible that hypoxia-induced erythrocyte Sphk1 activity regulates glucose metabolism. To test this hypothesis, we conducted glucose flux experiments using isotopic ^13^C_1,2,3_-glucose to trace how much glucose is metabolized to glycolysis and PPP, respectively, in cultured primary erythrocytes isolated from WT and *Sphk1*^*−/−*^ mice under normoxia and hypoxia at different time points (for details see Methods). As shown in Fig, 4b, if ^13^C_1,2,3_-glucose is metabolized directly through glycolysis, ^13^C_3_-lactate will be generated; while if glucose is metabolized through PPP, ^13^C_2_-lactate will be produced, owing to the release of the first carbon atom of glucose in the form of CO_2_ during glucose catabolism at the oxidative branch of the PPP. Ratios of ^13^C_3_-lactate/^13^C_2_-lactate isotopologue are thus indicative of glucose fluxes to glycolysis over PPP. First, we found that ^13^C_3_-lactate/^13^C_2_-lactate ratios were significantly induced in cultured WT mouse erythrocytes under hypoxia compared with normoxia in a time-dependent manner (Fig. 4c), indicating that hypoxia promoted significant increases in metabolic switch of glucose fluxes towards glycolysis in cultured WT mouse erythrocytes and supporting our findings from *in vivo* human erythrocyte metabolomic profiling in response to high altitude (Fig. 4a). Unexpectedly, we found that ratios of ^13^C_3_-lactate/^13^C_2_-lactate isotopologue were also significantly induced in cultured WT mouse erythrocytes under normoxia in a time-dependent manner, implicating that other factors besides hypoxia likely involved in switch of glucose fluxes to glycolysis in WT mouse erythrocytes in culture system under normoxia (Fig. 4c). Thus, to determine if erythrocyte Sphk1 is an important hypoxia-responsive molecule responsible for hypoxia-induced glucose fluxes towards glycolysis relative to PPP, we normalized the ratios of ^13^C_3_-lactate/^13^C_2_-lactate isotopologue under hypoxia to normoxia in WT and *Sphk1*^*−/−*^ mouse erythrocytes, respectively. As expected, we found that ratios of ^13^C_3_-lactate/^13^C_2_-lactate isotopologue under hypoxia to normoxia was significantly induced ∼1.5-fold from 1 h until 6 h in cultured WT mouse erythrocytes (Fig. 4d). In contrast, the fold induction of the ratios of ^13^C_3_-lactate/^13^C_2_-lactate isotopologue under hypoxia to normoxia was significantly attenuated in cultured *Sphk1*^*−/−*^ mouse erythrocytes compared with WT mouse erythrocytes (Fig. 4d), indicating that hypoxia-induced switch of fluxes of glucose from PPP to glycolysis is compromised in cultured *Sphk1*^*−/−*^ mouse erythrocytes. Altogether, these data indicate that erythrocyte Sphk1 contributes to the regulation of the hypoxia-dependent metabolic switch that promotes glucose metabolic fluxes through glycolysis.

### High altitude induces human RBC glycolytic enzyme activity

It has been shown that metabolism of glucose by the glycolytic pathway is limited by the availability of glycolytic enzymes such as glyceraldehyde-3-phosphate dehydrogenase (GAPDH) in cytosol, since most of the rate-limiting glycolytic enzymes are bound to the membrane^34^,^35^,^38^ and partially inhibited under normoxia^38^,^39^. In hypoxia, deoxygenated Hb (deoxy-Hb) binds to the cytosolic domain of band 3 at the membrane level and displaces the glycolytic enzymes, which in turn relocate to the cytosol and become more active, increasing fluxes through glycolysis^36^,^37^,^38^. While the mechanisms underlying this replacement have long been investigated, our findings suggest that S1P may represent a key regulatory contributor to the oxygen-dependent metabolic modulation model. Therefore, we tested whether hypoxia-induced increase in erythrocyte Sphk1 activity and elevation of S1P affected the binding of deoxy-Hb to membrane, resulting in the release and activation of glycolytic enzymes from membrane. We thus measured erythrocyte cytosolic activity of GAPDH, a key enzyme in the glycolytic pathway that is known to be bound on the membrane at high oxygen saturation^39^, in human volunteers at sea level and at high altitude. Supporting our hypothesis and metabolomic profiling result, we found that erythrocyte cytosolic GAPDH activity was significantly increased in a time-dependent manner in response to high-altitude hypoxia compared with sea level (Fig. 4e). Thus, we validated our metabolomic profiling and showed that cytosolic activity of the glycolytic enzyme-GAPDH, is significantly elevated concurrently with S1P, 2,3-BPG and O_2_ release capacity in response to high-altitude hypoxia (Fig. 4f).

### Hypoxia-induced GAPDH activity is blunted in *Sphk1* ^*−/−*^ mice

**Figure 5:**
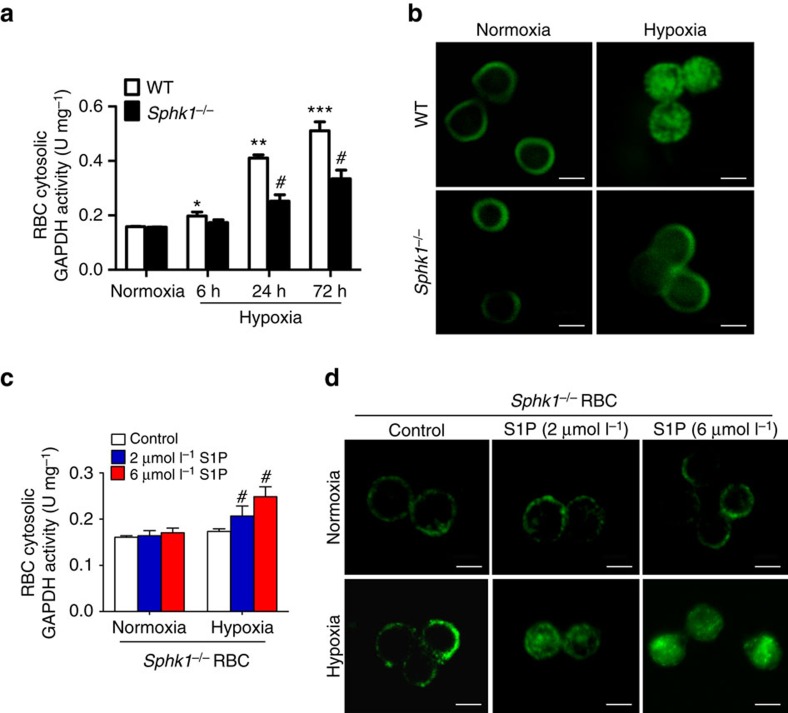
Sphk1-mediated production of S1P functions intracellularly underlying hypoxia-induced cytosolic GAPDH by inducing GAPDH release from membrane to cytosol. (**a**) Erythrocyte cytosolic GAPDH activity in WT and *Sphk1*^*−/−*^ mice treated in normoxia and hypoxia (10% O_2_) for different times. (**b**) Representative confocal images demonstrating GAPDH localization in erythrocytes from WT and *Sphk1*^*−/−*^ mice treated in normoxia and hypoxia for 72 h. (**c**,**d**) Cytosolic GAPDH activity (**c**) and representative confocal images (**d**) of primary cultures of *Sphk1*^*−/−*^ mouse erythrocyte pretreated with DMSO, 2 and 6 μmol l^−1^ S1P in normoxia and hypoxia (4% O_2_) for 6 h. Mean±s.e.m; *n*=4–6 per group; *P<0.05 versus normoxia, ^**^P<0.05 versus 6 h, ^***^P < 0.05 versus 24 h, ^#^*P*<0.05 versus control, Student's *t*-test and one-way ANOVA. Scale bar, 50 μM.

To determine whether the elevated erythrocyte Sphk1 facilitates glycolysis under hypoxia by regulating the release of membrane-anchored glycolytic enzymes such as GAPDH, we performed genetic mouse studies. We first measured cytosolic GAPDH activity in WT and *Sphk1*^*−/−*^ mice under normoxia and hypoxia at different time points. Consistent with our findings in humans, we found that hypoxia gradually induced cytosolic GAPDH activity up to three fold after 72 h exposure (Fig. 5a). However, hypoxia-induced elevation of erythrocyte cytosolic GAPDH activity was significantly reduced in *Sphk1*^*−/−*^ mice compared with WT mice (Fig. 5a). Meanwhile, we observed a significant increase of cytosolic GAPDH in WT mice erythrocytes after 72 h hypoxia treatment while to a much lesser extent in *Sphk1*^*−/−*^ mice using confocal microscopy (Fig. 5b). Thus, we validated our findings using human erythrocytes in mice and demonstrated that Sphk1 is essential for hypoxia-induced release of GAPDH from membrane to cytosol and subsequent elevated cytosolic GAPDH activity under hypoxia condition.

Next, to determine if function of Sphk1 is mediated by S1P surface receptors, we isolated erythrocytes from WT and *Sphk1*^*−/−*^ mice and pretreated them with exogenous S1P up to 250 nmol l^−1^ under normoxia and hypoxia conditions. S1P pretreatment up to 250 nmol l^−1^ had no effect on cytosolic GAPDH activity in cultured erythrocytes from WT under either normoxia or hypoxic conditions (Supplementary Fig. 5a). Moreover, S1P pretreatment could not rescue cytosolic GAPDH induction under both normoxia and hypoxia condition in cultured erythrocytes isolated from *Sphk1*^*−/−*^ mice (Supplementary Fig. 5b). Thus, these studies provided evidence that Sphk1 underlies hypoxia-induced GAPDH activity independent of S1P receptors in the erythrocytes.

It is known that erythrocytes can readily uptake exogenous S1P up to 5 μmol l^−1^ in an *in vitro* system^7^. Thus, to further assess if increased production of S1P due to hypoxia-induced Sphk1 activity functions intracellularly to induce membrane anchored GAPDH release to the cytosol and subsequently resulted in increased cytosolic GAPDH activity in the erythrocytes, we chose to test whether exogenous S1P at μmol l^−1^ concentrations known to be up-taken by erythrocytes can restore hypoxia-induced GAPDH activity by releasing membrane-anchored GAPDH to the cytosol in isolated *Sphk1*^*−/−*^ mouse erythrocytes. First, we found that under normoxia, pretreatment with S1P up to 6 μmol l^−1^ had no effect on cytosolic GAPDH activity in *Sphk1*^*−/−*^ erythrocytes (Fig. 5c). However, under hypoxia, S1P pretreatment at 2 μmol l^−1^ began to induce erythrocyte cytosolic GAPDH activity and reached higher level with 6 μmol l^−1^ S1P pretreatment (Fig. 5c). Consistently, confocal image analysis revealed that S1P treatment significantly induced translocalization of membrane-anchored GAPDH to the cytosol of *Sphk1*^*−/−*^ mouse erythrocytes in a dosage-dependent manner under hypoxia but not normoxia condition (Fig. 5d). These studies provided genetic evidence that S1P at μmol l^−1^ concentrations restored hypoxia-induced cytosolic GAPDH activity by promoting translocation of GAPDH from the membrane to the cytosol in *Sphk1*^*−/−*^ mouse erythrocytes. Altogether, our studies led us to conclude that erythrocyte Sphk1-mediated production of intracellular S1P triggers membrane-anchored/inhibited GAPDH release to the cytosol, subsequently promoting cytosolic GAPDH activity and, in this way, promoting fluxes through glycolysis, 2,3-BPG production and O_2_ release under hypoxia condition.

### S1P underlies hypoxia-induced GAPDH activity

**Figure 6:**
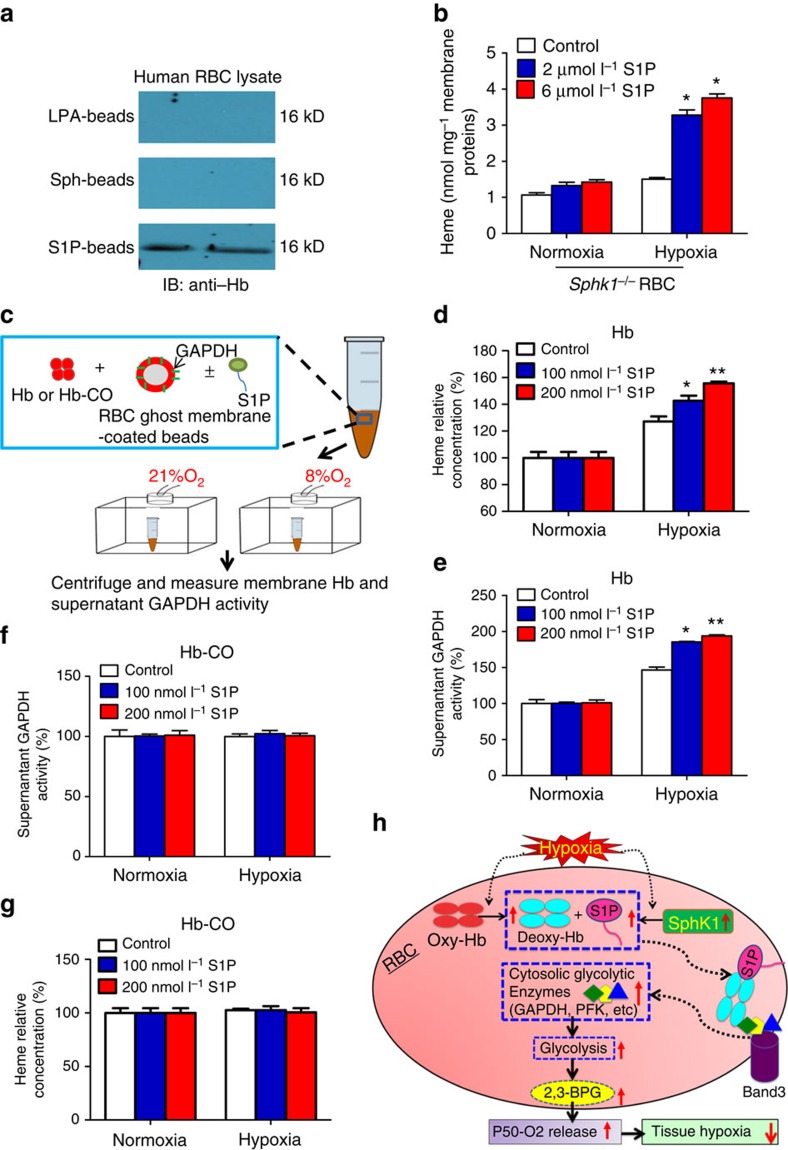
S1P promotes deoxy-Hb anchoring to the membrane and enhances GPADH release from membrane to cytosol only under hypoxia but not normoxia. (**a**) Pull-down of Hb by lysophosphatic acid, sphingosine and S1P beads from normal human RBC lysates. (**b**) Membrane heme concentrations in isolated *Sphk1*^*−/−*^ mouse erythrocyte pretreated with DMSO, 2 and 6 μmol l^−1^ S1P in normoxia and hypoxia (4% O_2_) for 6 h. (**c**) Schematic drawing illustrates functional experiments to monitor translocalization of GAPDH from membrane isolated from human erythrocytes. Hb binding to membrane (**d**) and GAPDH release from membrane to the cytosol (**e**) in human erythrocyte membrane ghost treated with Hb and different concentrations of S1P under normoxia and hypoxia. Hb binding to membrane (**f**) and GAPDH release from membrane to the cytosol (**g**) in human erythrocyte membrane ghost treated with Hb-CO and different concentrations of S1P under normoxia and hypoxia. (**h**) Working model: hypoxia-mediated elevation of erythrocyte Sphk1 activity increases the level of S1P, which binds to deoxy-Hb and facilitates binding of deoxy-Hb to membrane and release of GAPDH; increased cytosolic GAPDH accelerates glycolysis and shifts glucose metabolism in favour of 2,3-BPG production, which in turn leads to more O_2_ release to counteract tissue hypoxia. Mean±s.e.m; *n*=6 per group, *P<0.05 versus normoxia, ^**^P<0.05 versus 2 μmol l^−1^ or 100 nmol l^−1^, Student's *t*-test and one-way ANOVA.

Previous studies showed that organic phosphates (such as 2,3-BPG) can bind to Hb^40^,^41^,^42^,^43^,^44^. S1P, also an organic phosphate, is produced and stored at relatively high concentrations in RBCs. Thus, it is possible that S1P directly binds to Hb. To test this possibility, we first conducted pull-down assays using S1P immobilized to agarose beads to determine whether S1P directly binds to Hb. We found that S1P-beads, but not lysophosphatic acid beads or sphingosine (Sph) beads, successfully pulled down Hb from erythrocyte lysates of normal humans (Fig. 6a). This finding provides direct evidence that S1P directly interacts with Hb in the erythrocyte lysates. These findings raise an intriguing possibility that interaction of S1P with Hb can promote deoxy-Hb anchoring to the membrane thereby enhancing release of glycolytic enzymes (such as GAPDH) from membrane to the cytosol under hypoxic conditions. To test this hypothesis, we compared membrane-anchored Hb by measuring heme content in isolated *Sphk1*^*−/−*^ mouse erythrocytes treated with or without S1P at μM concentrations, which are known to increase intracellular S1P levels. Similar to S1P-mediated GAPDH release from the membrane (Fig. 5d), we found that S1P treatment only significantly increased membrane heme content under hypoxia but not under normoxia (Fig. 6b). Thus, we concluded that S1P treatment can restore hypoxia-induced deoxygenated Hb anchoring to the membrane and subsequent release of GAPDH from membrane to cytosol.

Finally, to validate above mouse findings, we conducted functional experiments to directly monitor (1) the alteration of membrane anchored Hb and (2) the translocation of GAPDH from human erythrocytes membrane under normoxia and hypoxia. We first prepared human erythrocyte ghost membranes and inversely coated on the silicon beads to expose inside out. Then, we incubated silicon beads coated with inverted erythrocyte ghost membranes with 100 μmol l^−1^ Hb in the absence or presence of S1P at 100 nmol l^−1^, to mimic the physiological molar ratio of Hb:S1P from 1,000:1 under different concentration of O_2_ ranging from fully oxygenated (21% O_2_) to hypoxia (8%). After 10-min incubation followed by brief centrifugation, supernatant GAPDH activity and membrane-anchored Hb were quantified, respectively (illustrated in Fig. 6c). We found that membrane anchored heme significantly increased under hypoxia compared with normoxia (Fig. 6d). Moreover, once Hb was fully oxygenated under normoxia condition (21% O_2_), S1P failed to induce oxy-Hb anchoring to membrane (Fig. 6d). However, under hypoxia condition, S1P further enhanced deoxy-Hb anchoring to the membrane (Fig. 6d). Next, we tested the functional kinetics of S1P on membrane-anchored deoxy-Hb under 8% O_2_ with different concentrations of S1P ranged from 0 to 200 nmol l^−1^. We found that S1P increased membrane-anchored deoxy-Hb but not oxy-Hb in a dose-dependent manner (Fig. 6d). Thus, these studies provide direct evidence that S1P forms a complex with Hb and promotes deoxy-Hb anchoring to the membrane in a hypoxia-dependent manner.

In parallel, we also monitored the release of GADPH from the membrane under normoxia and hypoxia, in presence of different doses of S1P as detailed above. We found that supernatant GADPH activity was significantly induced under hypoxia in a S1P dose-dependent manner in comparison with normoxia (Fig. 6e). In contrast, S1P had no effect on supernatant GAPDH activity under normoxia (Fig. 6e). Since CO was reported to highly stabilize R-state of Hb to inhibit glycolysis^53^, we examined if treatment with CO cancels effects of S1P on hypoxia-induced anchoring of Hb and GAPDH activity. In the CO-treated groups, we found that S1P had no effect on membrane anchoring of Hb (Fig. 6f) and GAPDH activity (Fig. 6g). Altogether, these data provide human evidence that S1P functions intracellullary as a hypoxia modulator promoting deoxy-Hb anchoring to the membrane and subsequently enhancing membrane-bound GADPH releasing to the cytosol, which in turn leads to increased cytosolic GAPDH activity under hypoxia.

## Discussion

Here we identified that S1P is significantly induced in humans following ascent to high altitude or mice exposed to hypoxia. Functionally, we demonstrated the beneficial role of Sphk1-dependent elevation of erythrocyte S1P by promoting 2,3-BPG production and O_2_ release to counteract tissue hypoxia. Mechanistically, we revealed that S1P functions intracellularly by binding directly to Hb, promoting deoxy-Hb anchoring to the membrane and subsequently enhancing the release of membrane-bound glycolytic enzymes to the cytosol. As such, increased erythrocyte S1P leads to increased metabolic fluxes through glycolysis to generate more 2,3-BPG and thereby promote O_2_ release to protect against tissue hypoxia (Fig. 6h).

Early studies showed that under hypoxia, deoxy-Hb binds to cytosolic domain of Band 3 (cdB3) on the membrane to cause release of glycolytic enzymes such as GAPDH from membrane to cytosol to enhance glycolysis and 2,3-BPG production^34^,^35^,^37^,^45^. However, the specific molecules mediating the binding of cytosolic deoxy-Hb binding to cdB3 and subsequent elevation of glycolysis under hypoxia remains unidentified. Intriguingly, non-biased metabolomics analyses on a rare sample set from a cohort of healthy human subjects exposed to high-altitude hypoxia has provided for the first time *in vivo* evidence of the presence of a hypoxia-dependent erythrocyte metabolic modulation including inducing glycolytic intermediates and reducing PPP intermediates. Extending human *in vivo* finding, we demonstrated for the first time that hypoxia directly induces switch of glucose fluxes through glycolysis from PPP in cultured mouse erythrocytes from WT mice but not in *Sphk1*^*−/−*^ mice. Because the T state of deoxy-Hb is stabilized by binding of organic phosphate^29^, it has long been speculated that anchoring of deoxy-Hb to the membrane may be mediated by binding of phosphate-containing lipophilic signalling molecules targeting the membrane. Here we report that SphK1-mediated elevation of S1P is a bioactive lipid that can bind directly to Hb and promote deoxy-Hb anchoring to the membrane, thereby promoting the release of membrane-bound/partially inhibited GAPDH to the cytosol, resulting in GAPDH activation, increased glycolytic fluxes, 2,3-BPG generation and, ultimately, increased oxygen off-loading capacity. It is known that binding affinity of 2,3-BPG to deoxygenated Hb is at μmol l^−1^ range, while to oxygenated Hb is at mmol l^−1^ (ref. 40). Thus, around 2–3 mmol l^−1^ elevation of 2,3-BPG under hypoxia condition observed in humans in high altitude and mouse in hypoxia chamber can likely bind to 2–3 mmol l^−1^ of oxygenated Hb (1:1 molar ratio) and in this way promote deoxygenation. Studies conducted in the 1960s showed that when humans descent from high altitude, 2,3-BPG levels dropped quickly to normal levels^29^. Notably, a recent study indicates that hypoxia-promoted erythrocyte Sphk1 activity is mediated by adenosine signalling via specific adenosine receptor A2B in a PKA and ERK1/2-dependent manner^46^. Therefore, it is possible that when back to normoxia, adenosine, a known hypoxia sensor, is reduced and turns off the elevated Sphk1 activity, which mediates the increased S1P production and subsequently reduces the S1P-based increase in 2,3-BPG levels.

Although S1P levels are the highest in mature erythrocytes among all the cell types^10^,^30^, the function of S1P in normal erythrocytes was a complete mystery prior to our study. Our human and mouse studies uncovered a previously unknown intracellular role of S1P in interacting with deoxy-Hb and regulating mature erythrocyte metabolism under hypoxic conditions. This finding presents a novel mechanism of lipid–protein interaction in the mouse and human erythrocyte for adaptation to hypoxia and reveals a novel key regulatory function of intracellular S1P in erythrocyte physiology and opens new therapeutic possibilities to enhance O_2_ release to counteract hypoxia at all conditions.

## Methods

### Human subjects

This study was conducted as part of the AltitudeOmics project examining the integrative physiology of human responses to hypoxia^47^. In brief, all procedures conformed to the Declaration of Helsinki were approved by the Universities of Colorado and Oregon Institutional Review Boards and the US Department of Defense Human Research Protection office. After written informed consent recreationally active sea-level habitants participated in the study. The participants were non-smokers, free from cardiorespiratory disease, born and raised at <1,500 m, and had not travelled to elevations >1,000 m in the 3 months prior to the investigation.

### Blood collection and preparation

Human and mouse blood were collected with heparin as an anti-coagulant and centrifuged at 2,000*g* for 5 min, followed by aspiration of plasma and white interface. Pelleted RBCs were washed once with 5 × volume of PBS before storing at −80 °C.

### Metabolomics profiling

RBCs (100 μl) and plasma samples (20 μl) were immediately extracted in ice-cold lysis/extraction buffer (methanol: acetonitrile: water 5:3:2) at 1:9 and 1:25 dilutions, respectively. RBCs were washed and samples were then agitated at 4 °C for 30 min and then centrifuged at 10,000*g* for 15 min at 4 °C. Protein pellets were discarded, while supernatants were stored at −80 °C prior to metabolomics analyses^48^. Ten microlitres of RBC extracts were injected into an UHPLC system (Ultimate 3000, Thermo, San Jose, CA, USA) and ran on a Kinetex XB-C18 column (150 × 2.1 mmol l^−1^ i.d., 1.7 μmol l^−1^ particle size; Phenomenex, Torrance, CA, USA) using a 3-min isocratic flow or a 9-min linear gradient (5–95% B in 9 min) at 250 μl min^−1^ (mobile phase: 5% acetonitrile, 95% 18 mil H20, 0.1% formic acid). The UHPLC system was coupled online with a QExactive system (Thermo, San Jose, CA, USA), scanning in Full MS mod (2 μ-scans) at 70,000 resolution in the 60–900 *m*/*z* range, 4 kV spray voltage, 15 sheath gas and auxiliary gas, operated in negative and then positive ion mode (separate runs). Calibration was performed before each analysis against positive or negative ion mode calibration mixes (Piercenet Thermo Fisher, Rockford, IL, USA) to ensure sub p.p.m. error of the intact mass. Metabolite assignments were performed using the software Maven (Princeton, NJ, USA), on conversion of raw files into mzXML format through MassMatrix (Cleveland, OH, USA). The software allows for peak picking, feature detection and metabolite assignment against the KEGG pathway database. Assignments were further confirmed against chemical formula determination (as gleaned from isotopic patterns and accurate intact mass), and retention times against a >750 standard compound library (SIGMA Aldrich, St Louis, MO, USA; IROA Tech, Bolton, MA, USA)^48^.

### S1P quantification

Validation and quantitative analyses for S1P were performed through multiple reaction monitoring (UHPLC-Q Exactive) on the precursor ion and transition fingerprints of the molecule, determined against commercial standard compounds C18-D-erythro-Sphingosine 1-phosphate (>95% pure—no. S9666, Sigma Aldrich, St Louis, MO, USA) and sphingosine-1-phosphate-d7 (>99% pure—SPH1PO4(D7)-11, catalogue no. 860659P—Avanti Lipids Polar Inc., Alabaster, AL, USA) within the linearity range, as determined through external calibration curves.

Relative quantification was performed by exporting integrated peak areas values for light and heavy isotopologue. Absolute quantification was determined according to the formula: (light)=ratios peak area (light)/(heavy) × (heavy) × dilution factor (9 for red blood cells, 25 for plasma). Results were imported into GraphPad Prism 5.0 (GraphPad Software Inc., La Jolla, CA, USA) for statistical analysis (one-way analysis of variance (ANOVA) with Tukey multiple column comparison test; significance threshold for *P* values <0.05).

### Mouse experiments

Eight to ten-week-old male and female C57BL/6 WT mice were purchased from Harlan Laboratories (Indianapolis, IN). Sphingosine kinase 1-deficient mice were initially acquired from Dr Richard L. Proia at the National Institute of Diabetes and Digestive and Kidney Diseases, NIH (Bethesda, MD) and bred in the University of Texas Health Science Center at Houston. All protocols involving animal studies were reviewed and approved by the Institutional Animal Welfare Committee of the University of Texas Health Science Center at Houston.

### Measurement of arterial haemoglobin-oxygen saturation in mice

Arterial oxygen saturation was measured as previously described^49^. Neck hair was shaved and a collar clip light sensor was set up on conscious mice. Arterial oxygen saturation was measured on conscious mice using the pulse MouseOx software analysis (STARR Life Sciences Corporation, Oakmont, PA, USA). Using pulse oximetry measurements of light absorption from the red and infrared LEDs, the MouseOx provides real-time percent oxygen saturation of functional arterial haemoglobin by utilizing.

### Isolation of total erythrocytes and *in vitro treatment*

Blood collected with heparin as an anti-coagulant was centrifuged at 2,000*g* for 5 min at room temperature, followed by aspiration of plasma and white interface. Packed mature RBCs were washed three times with culture media (F-10 nutrients mix, Life Technology) and re-suspended to 4% haematocrit. One millilitre of RBCs were added to each well of a 12-well plate and pretreated with different concentrations of S1P for 30 min (Sigma, USA) before transferring to normoxic or hypoxic conditions as indicated.

### Metabolic flux analysis

For the glucose flux experiment, RBCs were cultured in HEPES buffer with 6 mmol l^−1^ D-glucose-1,2,3-^13^C_3_ (Sigma Aldrich)^50^,^51^. RBCs were extracted and processed as described above. Flux analysis was performed by determining ^13^C_3_-lactate/^13^C_2_-lactate isotopologue ratios, determined by integrating the peak areas of isotopologues +2.0068 and +3.0102 of lactate in negative ion mode through the software Maven (Princeton University).

### Sphk1 activity assay

Erythrocyte Sphk1 activity was measured using previously described methods^52^,^53^ with a few modifications^46^. Briefly, RBCs were lysed in a pH 7.4 Tris-HCl buffer containing 1 mM EDTA, 1 mM β-mercaptoethanol, 0.3% Triton X-100, 50% glycerol and protease and phosphatase inhibitors. Then, the lysates were assayed using 250 μmol l^−1^ D-erythro-sphingosine in bovine serum albumin (0.4%) and [γ-^32^P]ATP (10 μCi, 20 mmol l^−1^) containing 200 mmol l^−1^ MgCl_2_. Lipids were extracted and then resolved by TLC on silica gel G60 with 1-butanol/methanol/acetic acid/water (80:20:10:20, v/v). The plates were then exposed to phosphor-imaging screening (Bio-Rad) and scanned for radioactive signals as indications of the amount of S1^32^P synthesized.

### Hypoxyprobe detection in multiple tissues *in vivo*

Tissue hypoxia levels were assessed by Hypoxyprobe immunofluorescence as described before^54^,^55^. Briefly, animals were administered Hypoxyprobe (Hypoxyprobe, Inc.) via intraperitoneal injection (60 mg kg^−1^ body weight). Thirty minutes after injection, tissues were harvested, fixed overnight in 4% buffered formalin and embedded in paraffin. Tissue sections were deparaffined, rehydrogenated and incubated with anti-Hypoxyprobe (rabbit anti-PAb2627AP, 1:200 dilution Hypoxyprobe, Inc.) overnight at 4 °C. Hypoxia signalling was detected by applying Alexa Fluor 488-conjugated donkey anti-rabbit IgG antibody (1:1,000 dilution, Life Technologies). Quantification of the fluorescent signalling was performed using the Image-Pro Plus software (Media Cybernetics, Bethesda, MD). The density of the fluorescence was measured. The average densities of 20 areas per samples were determined and the s.e.m. is indicated.

### 2,3-BPG analysis and erythrocyte oxygen release capacity (P50) measurement

2,3-BPG in 20 μl RBC pellete was isolated with 100 μl, 0.6 M cold perchloric acid on ice, vortexed and subsequently sonicated for 10 s with output 6 (W-220F, Heat Systems-Ultrasonic, Inc.). The homogenate was centrifuged at 20,000*g* for 10 min). A volume of 80 μl supernatant was transferred to a new tube and neutralized with 10 μl, 2.5 M K_2_CO_3_, then centrifuged at 20,000*g* for 5 min. A volume of 20 μl supernatant was used to quantify 2,3-BPG using a commercially available kit (Roche, Nutley, NJ)^56^,^57^ For human samples, arterial blood gases were measured and the Hill equation was used to calculate P50 (ref. 58. For mouse samples, 10 μl of whole blood aliquot were mixed with 4.5 ml Hemox Buffer (TCS Scientific Corporation, PA), 10 μl anti-foaming reagent ((TCS Scientific Corporation, PA) and 20 μl 22% BSA in PBS. The mixture was then injected into the Hemox Analyzer (TCS Scientific Corporation, PA) for measurement of oxygen equilibrium curve at the temperature of 37 °C.

### Irradiation and bone marrow transplant

The day before irradiation, recipient mice (12–14 weeks of age) were treated with neomycin at 2 μg ml^−1^ in drinking water, as described previously^9^. The next day, mice were exposed to 5 Gy irradiation by RS X-ray irradiator (Rad Source Technologies, Suwanee, GA). Four hours later, the mice were exposed to the same dose of irradiation. Bone marrow cells were isolated from femur of donor mice and injected retro-orbitally into irradiated recipient mice (1 × 10^6^ BMCs per mouse). After BMT, the mice were treated with 2 μg ml^−1^ neomycin in drinking water for 2 weeks. Mice were used 12–16 weeks later for experiments.

### Isolation of RBC cytoplasm and measurement of GAPDH activity

RBCs were lysed by freeze and thaw in 10 volumes of 5 mmol l^−1^ cold phosphate buffer (pH 8.0) and vortexed. RBC membrane was removed by centrifuged at 20,000*g* for 20 min at 4 °C. The supernatant was saved and used to measure cytosolic GAPDH activity by KDalert GAPDH assay kit (Life Technologies)^59^.

### Immunofluorescent staining of GAPDH in erythrocytes

Mouse erythrocytes were fixed with 100% ice-cold methanol for 10 min. The fixed cells were washed two times with PBS, blocked by 1% BSA in PBS (Blocking buffer, pH 7.4.) for 1 h at room temperature. Cells were incubated with monoclonal anti-GAPDH antibody (Sigma-Aldrich, 1:100 in blocking buffer) at 4 °C for overnight. The cells were washed three times with PBS, incubated with Alexa fluor 594 donkey anti-mouse IgG(H+L) (1:1,000 dilution) or Alexa fluor 488 donkey anti-rabbit IgG(H+L) (1:1,000 dilution) (Life Technologies) for 1 h at room temperature in dark, then washed three times and re-suspended in PBS. Cell-smear were made and dried in dark. The slides were mounted with cover glass by mounting medium (VECTASHIELD H-1400, Vector, CA). Pictures were taken under Zeiss LSM 780 confocal microscope (Carl Zeiss Inc., Jena, Germany).

### S1P beads pull-down assay

Two micrograms of total erythroctye lysate from normal individual was adjusted to 100 μl using lysis buffer (20 mM PIPES, 150 mmol l^−1^ NaCl, 1 mmol l^−1^ EGTA, 1% v/v Triton-X-100, 1.5 mmol l^−1^ MgCl_2_ and 1 mmol l^−1^ Naorthovanidate, 0.1% SDS, 1 × protease inhibitors (Roche Applied) pH 7.4). Approximately 100 μl of various lipids conjugated to agarose beads including S1P-agarose beads, lysophosphatic acid beads or sphingosine beads (Echelon Biosciences Inc., Salt Lake City, UT) were washed twice with lysis buffer. The lysates were incubated with beads overnight at 4 °C with constant gentle rotation. Protein-bound beads were washed by wash buffer (10 mmol l^−1^ HEPES pH 7.4, 150 mmol l^−1^ NaCl, 0.25% NP-40) for six times. Washed beads were added to 50 μl of 2 × Laemmli buffer (Sigma-Aldrich) and heated at 100 °C for 5 min. Beads were centrifuged at 5,000*g* for 5 min and supernatants (eluted proteins) were separated by SDS–polyacrylamide gel electrophoresis, transblotted to nitrocellulose membrane. Haemoglobin on the membrane was probed with anti-human haemoglobin antibody (Santa Cruz, CA). Immunoreactive bands were visualized by ECL using secondary antibodies conjugated with horseradish peroxidase and SuperSignal West Pico Chemiluminesence Substrate (Piere). Full scans of these immunoblots are available in Supplementary Fig. 6.

### *In vitro* reconstitution of ghost membrane assay

Nonporous silica beads with 3.15 μm diameter (Bangs Laboratories, IN) were pretreated with 6 ml of specific buffer (30% H_2_O_2_/30 % NH_4_OH/H_2_O: 1/1/5) and sonicated for 5 min, incubated at 60 °C for 30 min. The beads were washed with pH 5.5 millipore water for five times and then were washed with 5 mM PBS for three times. Human or mouse ghost cells were prepared as follows: heparin-blood was centrifuged at 2,400*g* for 5 min. The plasma and buffy coat were removed. The pellet was washed twice with PBS. The cells were lysed in 5 mmol l^−1^ phosphate buffer (pH 8.0), centrifuged at 18,000*g* for 15 min. The supernatant was removed and the pellet was washed in phosphate buffer for seven times to obtain ghost cells. The beads were coated by ghost cells to produce inside-out membrane (IOM). The IOM was washed six times with 5 mmol l^−1^ phosphate buffer (PB, pH 8.0). Packed 5 × 10^9^ IOM beads were added 100 μl, 5 mmol l^−1^ PB (pH 7.4) with 100 μM haemoglobin, varied concentration S1P. Beads were incubated at 37 °C for 10 min, centrifuged at room temperature for 1 min at 500*g*. The supernatant was transferred to new tube for GAPDH activity assay. Pellet beads were washed six times with PB (pH 7.4). Beads were added to 100 μl of concentrated formic acid (Sigma-Aldrich), vortexed for 5 min. The beads were centrifuged at 2,000 r.p.m. for 2 min. A volume of 80 μl of the supernatant was transferred to a new 1.5 ml tube and 400 μl, 5 M NaOH was added. The heme concentration was determined at 398 nm wavelength as described^60^ and normalized to protein concentration. Human Hb A was used as standards for heme assay. For hypoxia condition, PB was bubbled by 8% oxygen, 92% nitrogen for 10 min, then beads and PB were transferred to glove box. All the steps were performed in glove box until beads were ready for heme assay.

### Statistical analysis

All data were presented as mean±s.e.m, and analysed statistically using GraphPad Prism 5 software (GraphPad Software). The significance of differences among two groups was assessed using two-tailed Student's *t*-test. Differences between the means of multiple groups were compared by one-way ANOVA or two-way ANOVA, followed by a Turkey's multiple comparisons test. A *P* value <0.05 was considered significant.

### Data availability

We declare that the data supporting the findings of this study are available within the article and its Supplementary Information files and from the authors upon request.

## Additional information

**How to cite this article:** Sun, K. *et al*. Sphingosine 1-phosphate promotes erythrocyte glycolysis and oxygen release for adaptation to high-altitude hypoxia by. *Nat. Commun.* 7:12086 doi: 10.1038/ncomms12086 (2016).

## Supplementary Material

Supplementary Information Supplementary Figures 1-6

Supplementary Data Set 1 Raw Values
