# Supplementary material for: Sphingosine-1-phosphate promotes erythrocyte glycolysis and oxygen release for adaptation to high-altitude hypoxia
Source: Nat Commun. 2016 Jul 15;7:12086. doi: 10.1038/ncomms12086 (PMC4947158; doi:10.1038/ncomms12086)
Supplement: Supplementary Information — Supplementary Figures 1-6 [file ncomms12086-s1.pdf]

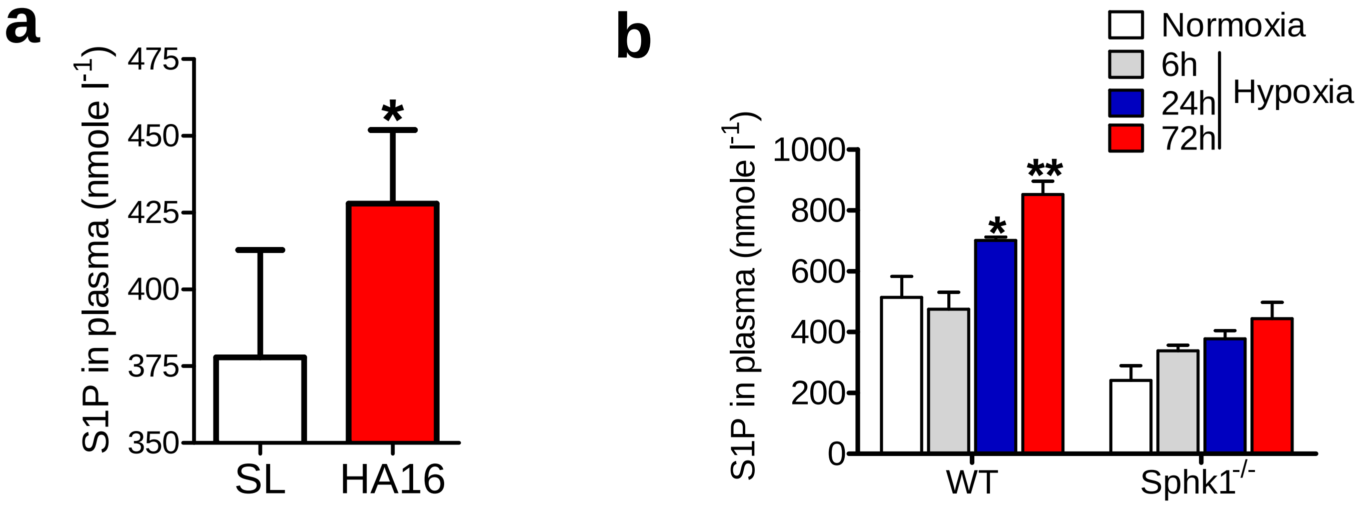

Supplementary Figure 1. Plasma S1P concentration increases in human and mice under hypoxia. (a) Plasma S1P concentration in human volunteers at sea level and after 16-day at high-altitude. (b) Plasma S1P concentration in WT and *Sphk1*$^{-/-}$ mice under normoxia and hypoxia at different time points. Mean ± s.e.m; N=16~21 for human samples; n=5 for mouse samples, *p<0.05 versus SL or 6h, **p<0.05 versus 24h, Student's t-test and one way ANOVA.

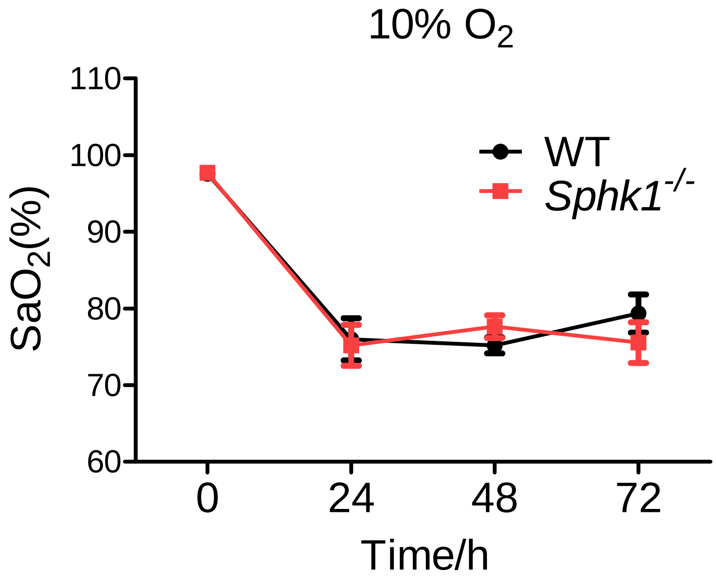

Supplementary Figure 2. No significant difference in arterial hemoglobin oxygen saturation (SaO_2) between WT and *Sphk1*$^{-/-}$ mice under normoxia and hypoxia up to 72 hours. Mean ± s.e.m; N=3~5 for each time point; No significant difference, Student's *t*-test.

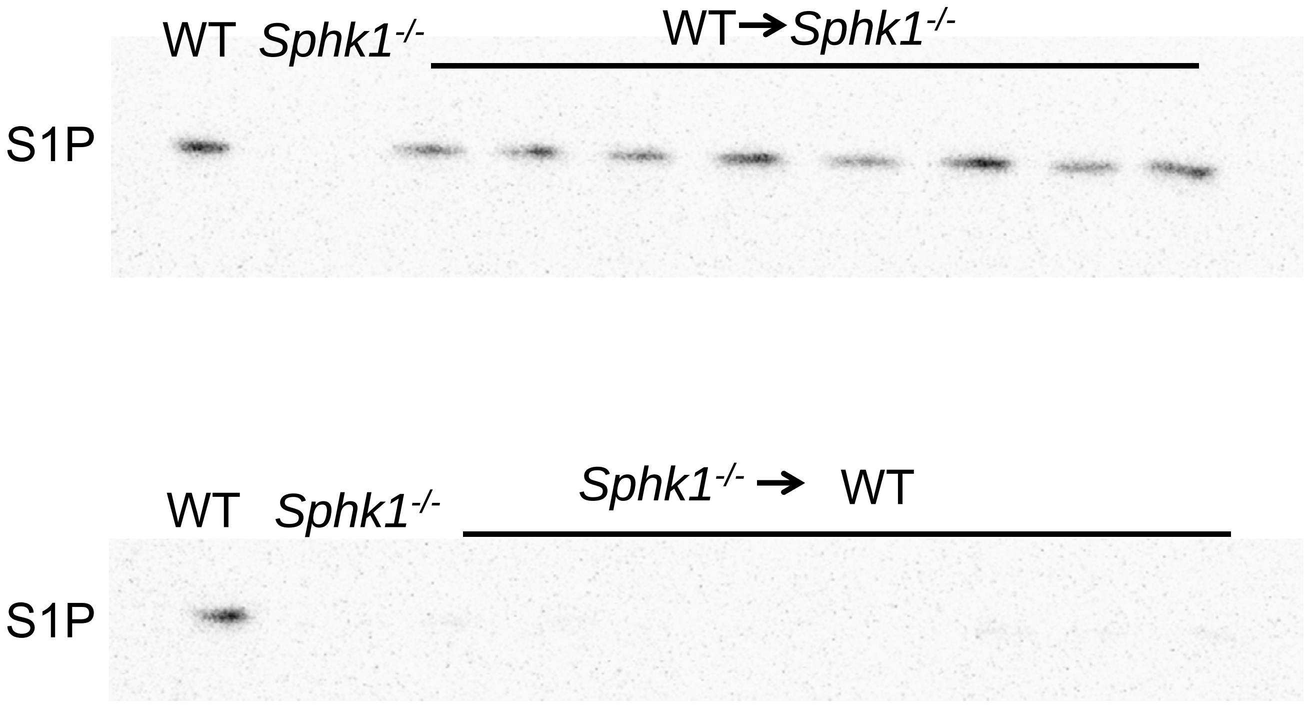

Supplementary Figure 3. Erythrocyte Sphk1 activity measurements in bone-marrow transplanted mice indicate a high chimerism with nearly 100% of circulating cells donor derived in receipts. Erythrocyte Sphk1 activity was assayed using D-erythro-sphingosine and [γ-^{32}P]ATP. Lipids were extracted and then resolved by TLC on silica gel G60 at the end of reaction. The TLC plates were then exposed to phospho-imaging screening (Bio-Rad) and scanned for radioactive signals as indications of the amount of S1P synthesized. The bands in the figure indicate ^{32}P-labeled S1P synthesize by Sphk1 in the erythrocytes.

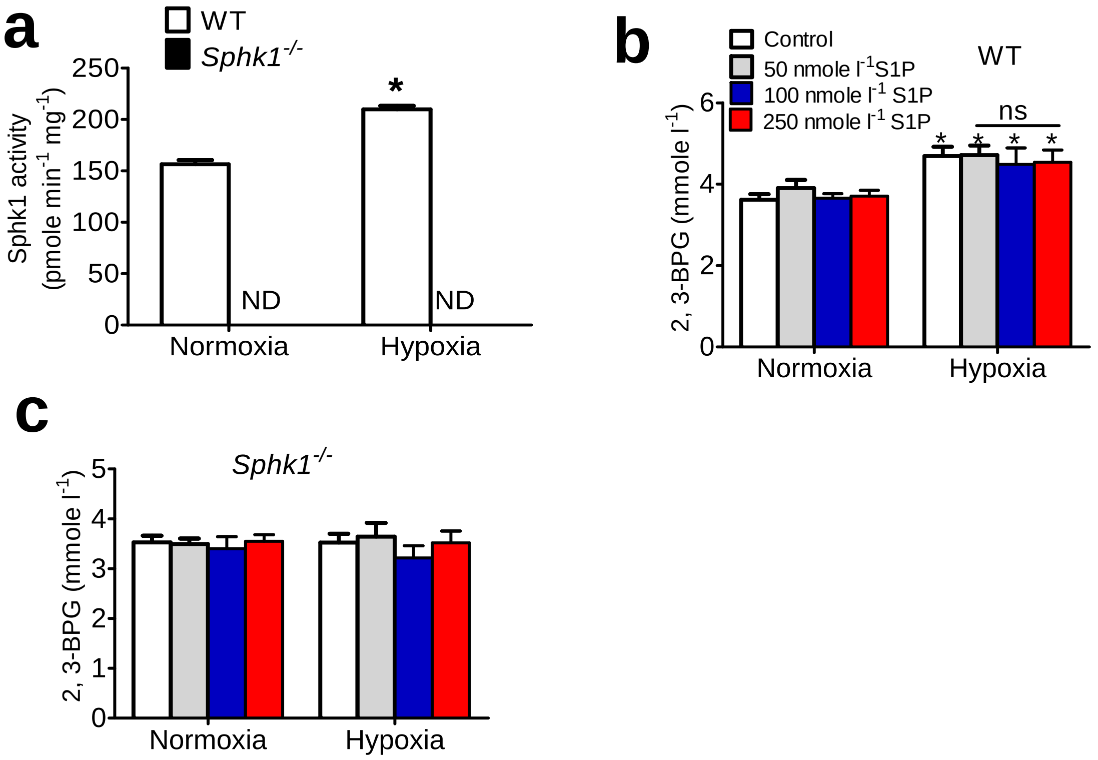

Supplementary Figure 4. Regulation of erythrocyte 2,3-BPG production by Sphk1-S1P is independent of S1P receptors. (**a**) Sphk1 activity in cultured erythrocytes isolated from WT mice under normoxia and hypoxia. (**b-c**) 2,3-BPG concentration in cultured erythrocytes isolated from WT (**b**) and *Sphk1*$^{-/-}$ (**c**) mice treated with vehicle or different doses of S1P under normoxia and hypoxia for 6 hours. Mean ± s.e.m; N=6~8 for each group; *p<0.05 versus normoxia, Student's *t*-test and one way ANOVA.

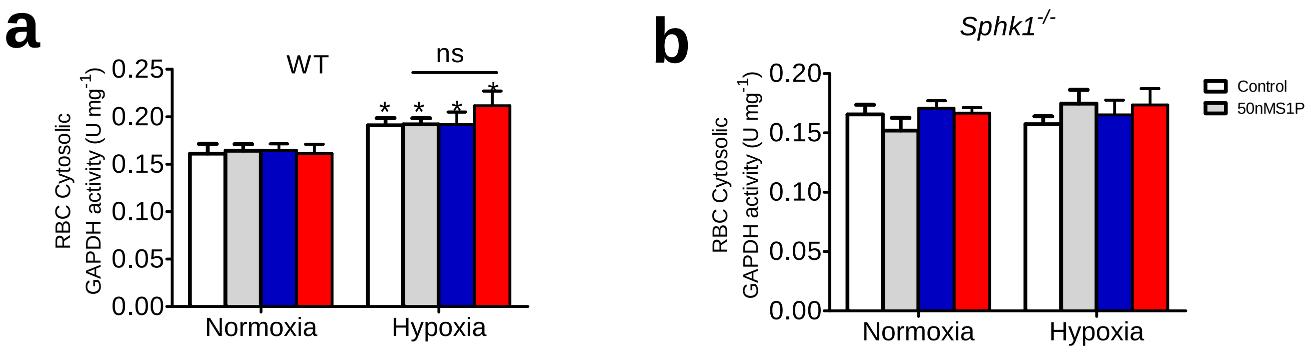

Supplementary Figure 5. Regulation of erythrocyte GAPDH localization by Sphk1 is independent of S1P receptors. (**a-b**) Cytosolic GAPDH activity in cutlured erythrocytes isolated from WT (**a**) and *Sphk1*$^{-/-}$ (**b**) mice treated with methonal or different doses of S1P under normoxia and hypoxia for 6 hours. Mean ± s.e.m; N=6~8 for each group; *p<0.05 versus normoxia, Student's *t*-test and one way ANOVA.

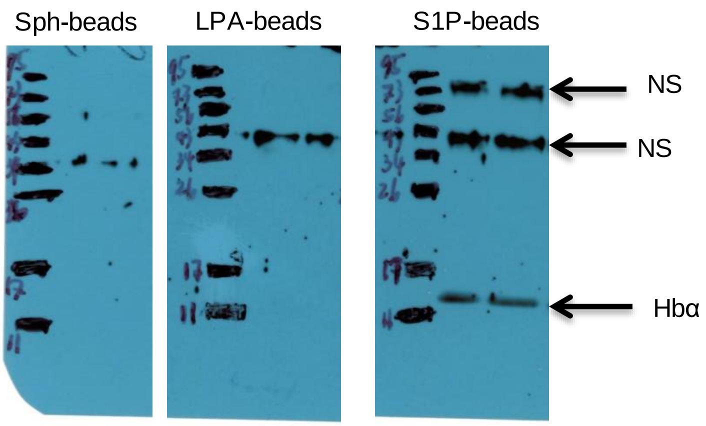

Supplementary Figure 6. This is the whole-blot pictures associated with Figure 6a. NS stands for non-specific band.
